# A Case of IgG1-Lambda Multiple Myeloma With Hyperviscosity Syndrome and Cryoglobulinemia: Identification of the Subclass Fraction by Immunoelectrophoresis and Immunofixation Electrophoresis

**DOI:** 10.7759/cureus.48253

**Published:** 2023-11-04

**Authors:** Kumiyo Tazoe, Naonori Harada, Kazuya Takemura, Mika Nakamae, Masayuki Hino

**Affiliations:** 1 Hematology, Osaka Metropolitan University Hospital, Osaka, JPN; 2 Hematology, Fuchu Hospital, Osaka, JPN; 3 Clinical Laboratory, Osaka Metropolitan University Hospital, Osaka, JPN; 4 Laboratory Medicine and Medical Informatics, Osaka Metropolitan University Hospital, Osaka, JPN

**Keywords:** plasmapheresis, cryoglobulinemia, igg subclass, hyperviscosity, multiple myeloma

## Abstract

Hyperviscosity syndrome (HVS) is a complication of monoclonal plasma cell tumors. The frequency of HVS depends on the type of monoclonal protein. Immunoglobulin M (IgM) is more closely associated with HVS than IgG, and among IgG subclass monoclonal proteins, IgG3 is most frequently associated with HVS. We herein report a 44-year-old woman with multiple myeloma (MM), HVS, and cryoglobulinemia. Her monoclonal protein and cryoglobulin were IgG1-lambda (λ). She developed HVS at a lower monoclonal protein level because of the properties of the IgG1-derived monoclonal protein and cryoglobulin. Our case highlights the fact that identifying the IgG subclass is useful in predicting the risk of complicating HVS.

## Introduction

Hyperviscosity syndrome (HVS) is an oncologic emergency in patients with abnormal immunoglobulin overproduction caused by monoclonal plasma cell tumors presenting with respiratory distress, neurological deficits, visual impairment, or mucosal bleeding [1]. Hyperviscosity syndrome occurs less frequently in patients with IgG multiple myeloma (MM) than in those with IgM gammopathy because the frequency of HVS complications depends on the molecular size of the monoclonal protein as well as the plasma concentration [1]. Furthermore, some reports state that even among IgG-type monoclonal plasma cell tumors, the amount of immunoglobulin required to cause HVS depends on the IgG subclass [2, 3]; however, there are few recent reports on this topic. We herein report a case of IgG-MM with HVS and cryoglobulinemia wherein the immunoglobulin and cryoglobulin subclasses were identified by immunoelectrophoresis (IEP) and immunofixation electrophoresis (IFE).

## Case presentation

A 44-year-old woman presented with worsening fatigue, headaches, visual impairment, and Raynaud's phenomenon. Whole-body computed tomography showed an increased bone marrow concentration but no osteolytic lesions or lymph node lesions. No bacteria or fungi were identified in blood cultures, and computed tomography at hospitalization did not show any infection focus. Laboratory findings were compatible with MM, including anemia, hyperproteinemia, and hypoalbuminemia (Table 1).

**Table 1:** Progress of laboratory tests after hospitalization PEX: plasma exchange; Bd: bortezomib and dexamethasone; DBd: daratumumab and bortezomib and dexamethasone; KRd: carfilzomib and lenalidomide and dexamethasone; BUN: blood urea nitrogen; β₂: beta 2;  κ: kappa; FLC: free light chain; λ: lambda

Day of hospitalization		Day 1	Day 1	Day 4	Day 4	Day 7	Day 14	Day 18	Day 19	Day 26	Day 28	Day 28	Day 33	Day 33
Parameter	Reference range	Before PEX	After PEX	Before PEX	After PEX	Bd start	DBd start	Before PEX	After PEX	Before PEX	After PEX	KRd start	Before PEX	After PEX
White blood cell (/μl）	4300-8000	11200				18100	20600					6600		
Red blood cell (×10⁴/μl)	395-495	237				296	271					256		
Hemoglobin (g/dl)	11.3-14.9	7.5				9.5	8.8					8.2		
Platelet (×10⁴/μl)	18.0-34.0	19.1				15.9	19.6					22.3		
BUN (mg/dl)	8-20	20				21	16					17		
Creatinine (mg/dl)	0.40-0.90	0.8				1.03	0.87					0.95		
Uric acid (mg/dl)	2.6-5.5	11.0				2.7	2.6					4.6		
Calcium (mg/dl)	8.8-10.1	8.6				9.9	10.2					10.5		
Total protein (g/dl)	6.6-8.1	15.2				12.0	12.3					10.2		
Albumin (g/dl)	3.5-5.0	1.7				3.0	2.3					3.2		
IgG (mg/dl)	870-1700	11700	5247	8429	2961	7287	8667	9013	4576	6859	2807	5677	6937	2132
IgA (mg/dl)	93-393	14	120	105	170	119	53	44	136	61	173	131	91	127
IgM (mg/dl)	50-269	10	53	48	62	47	29	26	44	26	57	46	37	65
β₂-microglobulin (mg/l)	0.80-2.00	10.87												
κ-FLC (mg/l)	3.30-19.40	5.7					1.5					1.3		
λ-FLC (mg/l)	5.71-26.30	2590					3140					2570		
FLC ratio	0.26-1.65	0.00					0.00					0.00		
Bence-Jones protein		Positive												
Cryoglobulin(mg/dl)		994												

**Figure 1:**
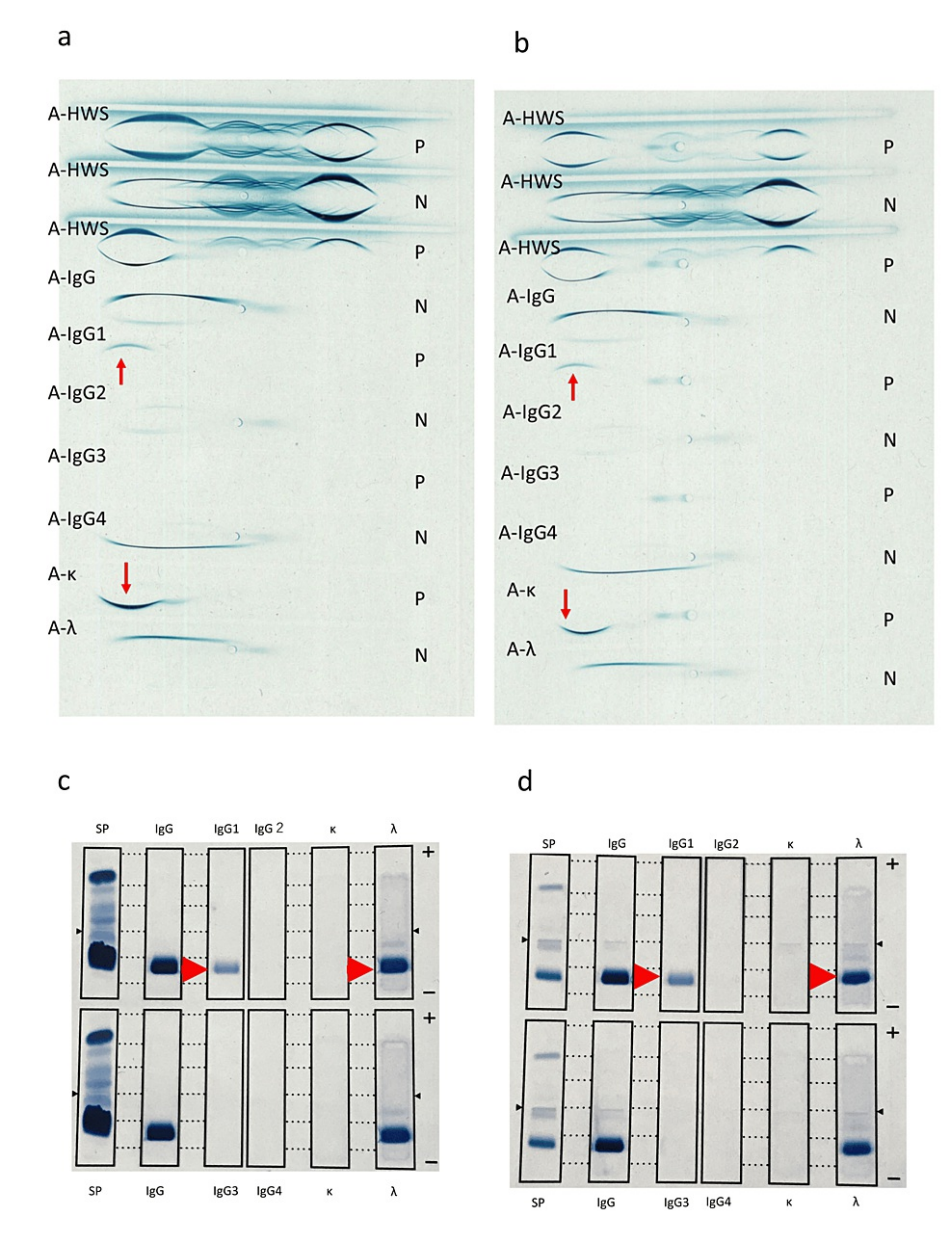
Immunoelectrophoresis and immunofixation electrophoresis (a) immunoelectrophoresis of serum proteins; and (b) serum cryoglobulin. The red arrow shows a significant monoclonal increase in IgG1, with an increased lambda immunoglobulin light chain. (c) immunofixation electrophoresis of serum proteins; and (d) serum cryoglobulin. The red arrow shows a dense band with anti-immunoglobulin G1 (IgG1) and anti-lambda serum. A-HWS: anti-human whole serum; A-IgG: anti-IgG serum; A-IgG1: anti-IgG1 serum; A-IgG2: anti-IgG2 serum; A-IgG3: anti-IgG3 serum; A-IgG4: anti-IgG4 serum; A-κ: anti-kappa serum; A-λ: anti-lambda serum; N: normal subject; P: patient

Serum IFE revealed an IgG-lambda (λ) monoclonal component. The patient was positive for serum cryoglobulin and a qualitative test for urinary Bence-Jones protein. In addition, we identified the subclass of monoclonal protein and cryoglobulin by IEP and IFE, which revealed that her monoclonal protein and cryoglobulin were IgG1-λ type (Figure 1).

The IgG1-derived monoclonal protein level was 8,510 mg/dl, and the IgG1 cryoglobulin level was 994 mg/dl.

Flow cytometry and chromosome tests of the bone marrow could not be performed because a bone marrow aspiration specimen was not obtained due to a dry tap. Immunostaining of a bone marrow biopsy revealed that 90% of the monoclonal plasma cells were CD38-positive, CD138-positive, CD56-negative, kappa (κ)-negative, and λ-positive. The patient was diagnosed with IgG1-λ-type multiple myeloma. According to the Revised International Staging System, her stage was III. She concurrently had HVS with retinal hemorrhaging. Therefore, she was admitted, and plasmapheresis (PEX) was immediately started with a short course of high-dose dexamethasone.

Her IgG level decreased from 11,700 to 5,247 mg/dl with PEX, and her headache improved. Bortezomib and dexamethasone therapy were started on day seven of hospitalization. As shown in Table 1, her IgG level continued to increase, and daratumumab was administered on day 14 of hospitalization. The PEX therapy was performed once a week to prevent her headache, which appeared when her IgG level increased to more than 6,000 mg/dl, even after the addition of daratumumab. Thus, the progressive disease was clinically confirmed.

Chemotherapy was switched to carfilzomib, lenalidomide, and dexamethasone (KRD) on day 28 of hospitalization. Even after switching to KRD therapy, the IgG level did not improve, and regular PEX therapy remained necessary to prevent clinical symptoms due to HVS. With frequent PEX use, she avoided any serious complications due to HVS, although multiple drugs were not effective. Thereafter, isatuximab, pomalidomide, and dexamethasone (IPd) were administered. The IPd therapy was effective, and the patient achieved a complete response.

## Discussion

**Figure 2:**
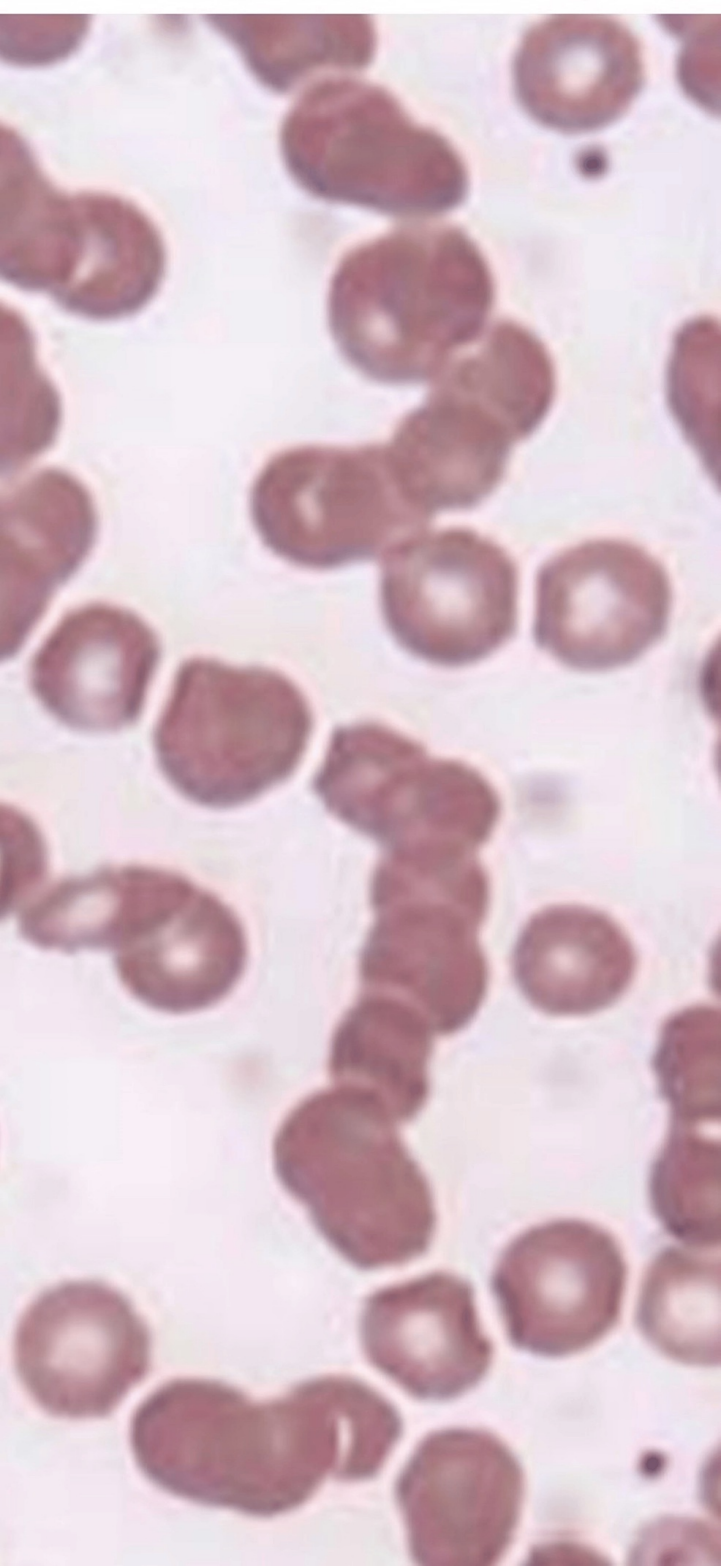
Peripheral blood smear Hyperviscosity syndrome often shows red blood cell rouleaux formation [5]

The main treatment for HVS is chemotherapy for monoclonal protein-producing diseases and PEX to temporarily reduce the amount of monoclonal protein. There are no clear criteria for the initiation or frequency of PEX, and it has been reported that PEX should be performed so that clinical symptoms do not appear [4]. Thus, predicting the appearance of symptoms is important for appropriate treatment. Hyperviscosity syndrome often shows the formation of red blood cell rouleaux in peripheral blood smears (Figure 2) [5].

Hyperviscosity syndrome is attributed to increased blood cells and increased plasma viscosity. Plasma proteins determine the viscosity level, and their three-dimensional structure plays an essential role [1]. While spherical proteins, which rotate through the plasma, have almost no effect on viscosity, linear proteins can raise the viscosity by spinning end over end [1]. The linear proteins are mainly fibrinogen and immunoglobulin; thus, immunoproliferative disorders often lead to HVS [6].

The rate of HVS complications depends on the intrinsic viscosity and molecular weight of immunoglobulins. The incidence of symptomatic HVS is associated with monoclonal protein-producing tumors in the order of IgM>IgA>IgG because of differences in molecular size. Among these monoclonal proteins, IgG-MM has different characteristics depending on its subclass; therefore, the frequency of HVS complications differs among subclasses [3]. In patients with IgG-MM, the ratio of IgG subclasses was IgG1 (66%) >IgG2 (18%) >IgG3 and IgG4 (4%) [7]. However, in a study in which IgG-MM patients with HVS were analyzed for IgG subclasses, the most common subclass was IgG1 (76.4%), and the second-most common subclass was IgG3 (15.4%) [3]. Thus, these previous reports indicated that a relatively high proportion of IgG3-MM caused HVS. This is because IgG3-derived monoclonal proteins have the unique characteristic of developing concentration- and temperature-dependent aggregates [3,8].

Similarly, type I IgG cryoglobulinemia, which appears secondary to multiple myeloma and can cause HVS, has different characteristics depending on the IgG subclass [9]. Cryoglobulins are immunoglobulins that precipitate at low temperatures and re-dissolve at body temperature [9]. According to a previous report, IgG causes cryoglobulinemia frequently by forming an immune complex and activating complement; among IgG subclasses, IgG1 and IgG3 are reported to form a larger immune complex and to more easily activate complement than others [10]. Furthermore, IgG1-derived cryoglobulin is reportedly likely to cause serious vasculitis [11]. In other words, IgG1-derived cryoglobulin is considered to have an affinity for the microvascular system. Therefore, the identification of IgG subclasses of IgG-MM and IgG-cryoglobulinemia may be useful for predicting the future risk of HVS.

However, most studies that mentioned IgG subclasses in IgG-MM with HVS were published in the 1970s. Since then, no reports have described the identification of subclasses using IEP and IFE. In our case, we performed IEP and IFE of IgG subclasses to understand the pathology in greater detail and identified the subclass as the IgG1-λ type. Our patient had the most common IgG subtypes. However, interestingly, her IgG level when HVS symptoms appeared was lower than the previously reported monoclonal protein concentration of IgG-MM with HVS, except for the IgG3 subtype [12].

The present patient had IgG1-derived cryoglobulin and developed HVS in the winter. As a result, she developed HVS at a lower IgG level than that previously reported due to IgG1-derived cryoglobulinemia, which is likely to obstruct the microvasculature. In the present case, detailed information on monoclonal proteins and cryoglobulin was able to be obtained using IEP and IFE.

In the future, in similar cases where the amount of monoclonal protein is atypical for the development of HVS, the presence of cryoglobulins might contribute to HVS. Looking for the presence of cryoglobulinemia and measuring serum viscosity is beneficial for appropriate treatment. Furthermore, IgG-derived cryoglobulins have different characteristics depending on their subclass, so identifying the cryoglobulin subclass using IEP or IFE may be useful for understanding the pathology.

## Conclusions

The appropriate duration of PEX for HVS may depend on the type of monoclonal protein. Furthermore, IgG1-type monoclonal proteins occasionally lead to cryoglobulinemia. Our case highlights the notion that understanding the features of monoclonal proteins, including the IgG subclass, may help hematologists determine appropriate supportive care for patients with MM.
